# The Effect of Z-Ligustilide on the Mobility of Human Glioblastoma T98G Cells

**DOI:** 10.1371/journal.pone.0066598

**Published:** 2013-06-21

**Authors:** Jun Yin, Chengyuan Wang, Aaron Mody, Lin Bao, Shen-Hsiu Hung, Spyros A. Svoronos, Yiider Tseng

**Affiliations:** 1 Department of Chemical Engineering, University of Florida, Gainesville, Florida, United States of America; 2 Research Center of Medical Chemistry and Chemical Biology, Chongqing Technology and Business University, Chongqing, China; 3 National Cancer Institute - Physical Science Oncology Center, Gainesville, Florida, United States of America; 4 Institute for Cell Engineering and Regenerative Medicine, Gainesville, Florida, United States of America; University of Pittsburgh, United States of America

## Abstract

Z-ligustilide (LIG), an essential oil extract from *Radix Angelica sinensis*, has broad pharmaceutical applications in treating cardio-vascular diseases and ischemic brain injury. Recently, LIG has been connected to Glioblastoma multiforme (GBM) because of its structural similarity to 3-n-alkyphthalide (NBP), which is specifically cytotoxic to GBM cells. Hence, we investigated LIG’s effect on GBM T98G cells. The study shows that LIG can significantly reduce T98G cells’ migration in a dose-dependent manner. Furthermore, the attenuation of cellular mobility can be linked to the activity of the Rho GTPases (RhoA, Rac1 and Cdc42), the three critical molecular switches governing cytoskeleton remodeling; thus, regulating cell migration. LIG significantly reduces the expression of RhoA and affects in a milder manner the expression of Cdc42 and Rac1.

## Introduction

*Radix Angelica sinensis* (RAS) has been considered a medicinal plant and applied to alleviate various disease syndromes in traditional Chinese medicine for over a thousand years. Approximately, more than 70 compounds have been currently identified in RAS, including phthalide dimers, organic acids and their derivative esters, polyacetylenes, vitamins, amino acids, and essential oils [1]. Among the essential oils of RAS, Z-ligustilide (LIG) is one of the most active components and has been characterized for more than 40 years.

LIG can inhibit the proliferation and cell cycle progression of vascular smooth muscle cells, associated to basic fibroblast growth factor stimulation, through the reduction of reactive oxygen species and/or the suppression of the MAPK pathway [2]. LIG also inhibits vasoconstriction induced by norepinephrine bitartrate and calcium chloride on rat abdominal aorta segments [3]. Hence, LIG is considered to be an effective agent to reduce vascular resistance; thereafter, increase blood flow and enhance microcirculation to prevent cardiovascular diseases**,** including atherosclerosis and hypertension [4], [5].

Meanwhile, LIG has an analgesic effect on rats and a concentration-dependent anti-inflammatory effect on lipopolysaccharide-activated rat microglia without cytotoxicity [6], [7]. LIG is also known to have a protective effect against ischemic brain injury caused by the failure of regular blood supply to local brain tissue in the central nervous system (CNS) [8]. LIG decreases the level of malondialdehyde, a product of lipid peroxidation, and increases the activity of antioxidant enzymes, fostering an anti-apoptotic effect that reduces cerebral infarct volumes and improves neurobehavioral deficits [9].

**Figure 1:**
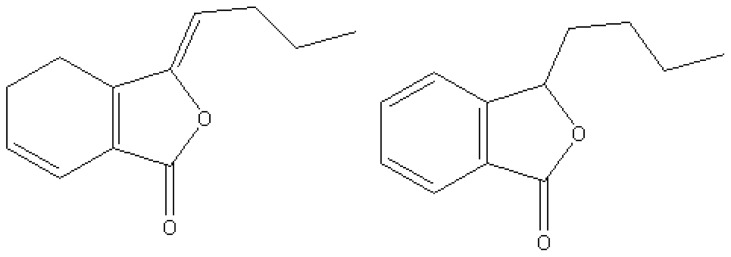
Chemical Structures of Z-Ligustilide (LIG) and 3-n-Butylphthalide (NBP).

The structure of LIG is similar to that of n-butylidenephthalide (NBP) (Fig. 1), which has also been demonstrated to possess activity to reduce inflammation and hepatotoxicity as LIG does [1]. A recent study has revealed that NBP could suppress the growth of Glioblastoma Multiforme (GBM) cells both *in vitro* and *in vivo* via cell cycle arrest and apoptosis [10]. GBM is the most common and aggressive malignant primary brain tumor, represents 50% of all gliomas and has the worst prognosis of any CNS malignancy despite the progression of existing diagnosis methods and treatments [10]. The unrevealed rapid invasion mechanism of GBM presents a great challenge to accurately predict the development of GBM and efficiently treat it. As a derivative of NBP, LIG might have similar pharmaceutical effects on GBM diseases; therefore, the pharmaceutical outcome of LIG treatment of GBM is worth investigating.

The most common assessments for drug effects on cells are endpoint evaluations, such as the induction of apoptosis and the change in cell proliferation. However, other beneficial drug effects might exist and can be tested through non-conventional methods. In this study we explored the effect of LIG treatment on T98G cells – not only using endpoint assessments, but also by evaluating the changes in cell migration patterns, one of the most critical cell activities of cancer metastasis. Cell migration patterns were assessed at both the cellular and the molecular level. The three Rho GTPases (RhoA, Rac1 and Cdc42) are the main molecular switches that govern cytoskeletal remodeling to regulate cell migration [11], and these Rho GTPases are usually linked to cancer by changes in their expression profiles rather than by mutations [11]. Hence, the expression levels of these three proteins were evaluated via Western blotting.

## Materials and Methods

### Cell Culture and Z-ligustilide Preparation

T98G cells were obtained from ATCC (American Type Culture Collection, Manassas, VA) and cultured in EMEM (Mediatech, Manassas, VA), supplemented with 10% fetal bovine serum (Hyclone Laboratories, Logan, UT). Cell cultures were maintained in a 5% CO_2_ incubator with routine passing every 2 ∼ 3 days. Cells were transferred onto glass bottom dishes (In Vitro Scientific, Sunnyvale, CA), pre-treated with 0.01% poly-L-lysine (Sigma-Aldrich, St Louis, MO) and 20 µl/ml fibronectin (BD Biosciences, Bedford, MA), for image acquisition.

LIG was isolated by silica gel column chromatography from the *Angelica sinensis* essential oil, which was extracted using supercritical-CO_2_ fluid. LIG was then identified by ^1^H and ^13^C nuclear magnetic resonance spectrometry and electron impact ionization mass spectrometry as 99.8% pure (density  = 0.979±0.005 g/ml). Because it is insoluble in water, isopropanol (IPA) was used as an effective solvent. LIG was first diluted 1,000-, 2,500-, 5,000- and 10,000-fold in IPA, and then applied to T98G cell cultures at a further 200-fold dilution to reach final LIG concentrations of 25, 10, 5 and 2.5 µM, respectively.

### Wound-like Gap Closure Assay

Wound-like gap closure (or wound healing) assays were performed on 6-well pre-treated glass bottom dishes (In Vitro Scientific). Cells were loaded at a concentration of ∼ 5×10^5^ cells/ml, and grown to confluence. A glass pipet was used to physically scratch a ∼ 400 µm wound gap in the monolayer of cells. Samples were then washed with HBSS (Mediatech Inc.) and then incubated in fresh culture media before the following additions: 1) mock (negative control); 2) 0.5% IPA; 3) 2.5 µM LIG; 4) 5 µM LIG; 5) 10 µM LIG; and 6) 25 µM LIG. Here, the sample with 0.5% IPA also served as a control to monitor the effect of the solvent of LIG at the same applied concentration on cells. Cell motility was recorded using a Nikon TE200E microscope system (Nikon Instruments, Melville, NY), equipped with a 10× objective lens and an EMCCD camera (Roper Scientific, Tucson, AZ). Six positions along the scratched areas under each condition were recorded at several time points during a 14-hour period for further analysis.

### Quantification of Wound-like Gap Closure Process

A gap index (*GI*) was employed using the ratio of remaining area to the original wound gap area utilizing a custom algorithm coded in Matlab (The Mathworks, Natick, MA). Each 1000-pixel×1000-pixel image at the initial time, *t_0_*, was divided into 2500 20-pixel×20-pixel boxes and the average intensity of each box was obtained. Twenty boxes without cells were picked to determine the average background intensity, and then three standard deviations above the average background intensity was set as a threshold value. The *GI* of selected areas at time *t* was calculated by the following equation:
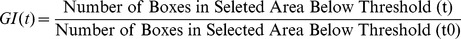



### Cell Proliferation and Apoptosis Assay

T98G cells at a concentration of ∼1×10^5^ cells/well were plated in 12-well culture dishes (Corning Inc., Corning, NY). Cells in media with either no treatment, 0.5% IPA or 5 µM LIG were counted by a hemocytometer at different time points until cell concentration was doubled. Triplicate tests at each treatment condition were conducted, and each of the triplicates was measured 3 times. Cell counts vs. time were plotted and fitted into exponential curves.

The APO-DIRECT KIT (BD Biosciences) was used to assess the apoptotic state of T98G cells with and without the presence of 0.5% IPA and 5 µM LIG at high and low cell concentrations corresponding to wound-like gap closure assay and single cell assay, respectively. T98G cells treated with 8µM camptothecin (Sigma-Aldrich) for 20 hours were used as a positive control. Samples were assessed using an ACCURI C6 Flow Cytometer (BD Biosciences).

### Single Cell Assay and Data Analysis

The pEGFP plasmids (BD Biosciences) were introduced into T98G cells at approximately 50%–70% confluence by transfection. The transfected cells were re-plated on fibronectin coated 6-well glass bottom plates (In Vitro Scientific) at a concentration low enough to avoid cell-cell interactions. After 5 hours of incubation with 0.5% IPA or 5 µM LIG, single cells expressing green fluorescence protein were monitored every minute for 1 hour with a 20× objective lens under a UV light source (X-Cite 120 PC fluorescent light source, EXFO, Ontario, Canada). Cell movement videos were analyzed using the MATLAB software image processing toolbox.

### Western Blotting

Samples were prepared by seeding T98G cells at high (∼5×10^5^ cells/ml) and low (∼5×10^3^ cells/ml) concentrations, corresponding to wound-like gap closure assay and single cell assay, respectively. Cells prepared at high concentration were incubated for 20 hours with no treatment, 0.5% IPA or 5 µM LIG to probe the individual activities of the Rho GTPases in the wound-like gap closure process under different treatment conditions. Cells at low concentration were incubated for 5 hours with no treatment, 0.5% IPA or 5 µM LIG to probe the activities of the same proteins in single cell assay conditions. Also, low-density cells with the same treatments were incubated for 20 hours for cross-comparison purposes.

Each individual culture sample was suspended in 300 µl lysis buffer (Cytoskeleton, Denver, CO) with 1% of protease inhibitors (Cytoskeleton) and homogenized on ice. After centrifugation at 4°C, the supernatants of the cell lysates were separately collected for protein detection. Total protein concentration of a supernatant was measured using the Coomassie Plus Protein Assay (Thermo Scientific, Waltham, MA) and ∼ 8 µg of sample was loaded on a 12% polyacrylamide gel for electrophoresis, followed by transference onto a PVDF membrane (BIO-RAD, Hercules, CA).

Individual Rho GTPases were detected with the specific monoclonal antibody against Rac1 (Millipore, Billerica, MA), RhoA (Santa Cruz Biotech Inc., Dallas, TX) or Cdc42 (BD biosciences). Here, GADPH (Santa Cruz Biotech Inc.) activity was used as a baseline control for all the blotting. Results of western blotting were quantified using ImageJ (NIH, Bethesda, MD), treating proteins in untreated T98G cells as reference.

## Results

### LIG Exhibits a Cellular Effect Hindering the Migration Activity of T98G Cells

**Figure 2:**
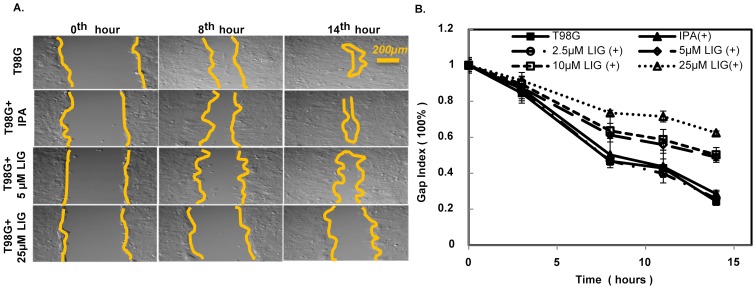
The addition of LIG to T98G cell cultures reduced migration capacity. **A**) Images of the wound-like gap closure process of T98G cells under mock, 0.5% isopropanol (IPA), 5 µM LIG, and 25 µM LIG (top to bottom) are presented at the 0^th^, 8^th^ and 14^th^ hour. The yellow lines indicate the approximate boundary between cell-inhabited and cell-free (central) regions of each image. Scale bar: 200 µm. **B**) The result of a quantitative analysis of the gap closure of T98G cells under no treatment, 0.5% IPA, and 4 concentrations (2.5, 5, 15 and 25 µM) of LIG treatment is shown. The gap index is the percentage of original wound gap that remains cell-free. The data are the mean gap index results of 6 experiments with error bars representing the standard deviation (also refer to Fig. S1).

To explore whether LIG treatment affects the migration of T98G cells, LIG was diluted to different concentrations in IPA and applied to T98G cell cultures immediately after the wound-like gap was created. The progression of wound-like gap closure was then recorded and analyzed. The results suggest that wound closure capacity decreases in the presence of LIG but not for IPA treatment (Fig. 2A). Moreover, this LIG effect was dose dependent (Fig. 2 B). Based on these results, 5 µM is the lowest dose of LIG that has significant effect on cell migration; so we chose this dosage for further study.

### The Migration-hindering Effect was not caused by Reduction in Cell Proliferation Rate or Apoptosis

**Figure 3:**
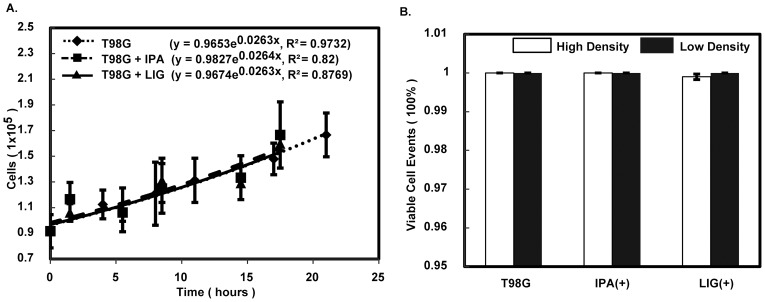
Neither cell proliferation nor apoptosis explain the attenuation of wound-like gap closure in the presence of LIG. **A**) Comparative growth of T98G cells under no treatment, 0.5% IPA and 5-µM LIG treatment is shown. Each population of T98G cells was counted every 3 hours for 24 hours. The average number from 9 assessments at each time point was fit to an exponential curve to obtain the growth rate under each treatment condition. Error bars represent the standard deviation. **B**) The result of apoptosis assays for both high and low density of T98G cells is shown. The bars represent the average between two measurements of viable cell percentages under no treatment, 0.5% IPA, and 5-µM LIG treatment, respectively.

Changes in cell proliferation alter the cell population and may affect the wound-like gap closure process; hence, the effect of LIG at 5 µM on the proliferation rate of T98G cells was investigated. A hemocytometer was employed to count cell populations with or without the presence of LIG over a 20-hour period. Cell populations over the monitored time periods were fit by exponential curves to obtain the growth equations and regression coefficients (Fig. 3 A). Population growth rates suggest that LIG at the tested concentration did not lead to any detectable difference on cell proliferation (specific growth rates: 0.0263 hr^−1^ for T98G, 0.0264 hr^−1^ for T98G+IPA and 0.0263 hr^−1^ for T98G+LIG). Hence, the reduction of wound-like gap closure capacity of T98G cells was not due to a change in cell proliferation.

Cell apoptosis could also affect the cell population during the wound-like gap closure process. Hence, the ability of LIG at the working concentration to induce cell apoptosis was examined on T98G cells of both low and high density. The percentage of cell viability (2 trials) indicated that LIG does not induce T98G cell apoptosis at both high and low cell concentration (Fig 3B; also see Figure S2). Thus, the LIG effect that hinders the wound-like gap closure process was not due to induction of apoptosis.

### LIG Treatment Affects Single T98G Cell Migration

Cell-cell interactions can also affect the progression of wound-like gap closure. The interplay between the signaling pathways governing cell migration and cell-cell interaction has been investigated intensely, especially the topic of endothelial-to-mesenchymal transition (EMT) related to cancer metastasis [12]. Therefore, cell-cell interaction should not be overlooked as a potential factor that influences wound-like gap closure. To that end, the effect of LIG on the mobility of individual T98G cells was studied.

**Figure 4:**
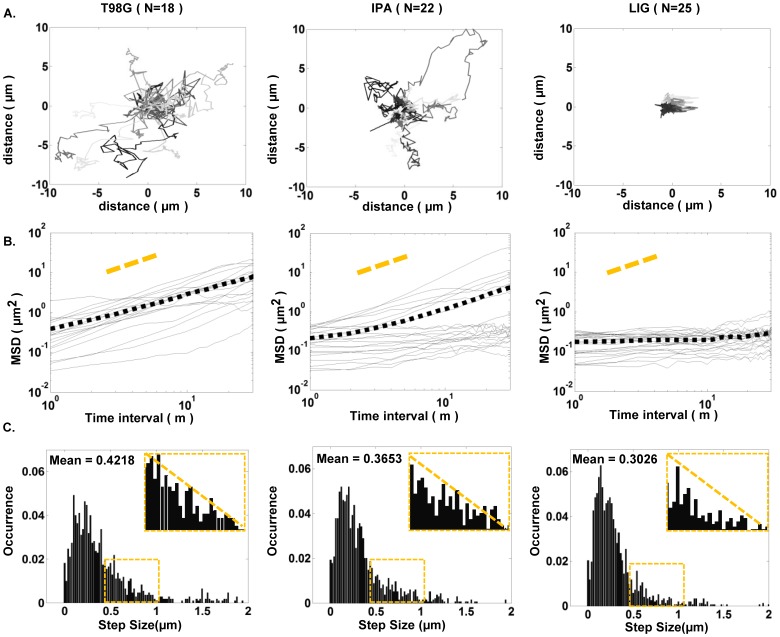
LIG treatment reduced T98G single cell migration capacity. **A**) Trajectories of T98G cells indicate that the presence of LIG could drastically hinder cell migration, and the addition of 0.5% IPA has a minor effect. **B**) The mean square displacement (MSD) vs. time interval is shown as a log-log plot, demonstrating that LIG treatment significantly decreases the slope of the MSD of T98G cells as compared to control conditions. The mean MSD value for each condition is plotted as a dotted black line. **C**) Histograms of the one-minute step sizes within one hour suggest a reduction of the translocation of T98G cells under LIG treatment since the occurrence of larger step sizes is greatly reduced.

Fluorescence images of single T98G cells expressing green fluorescence protein (GFP) were captured every minute for one hour to track their movement after the cells were subjected to no treatment, 0.5% IPA or 5 µM LIG for 5 hours. In each image, the cell centroid was determined and the cell centroid displacements (CCD) between images were calculated and combined to form single cell trajectories (Fig. 4 A). The lengths of T98G cell trajectories indicate that 0.5% IPA might also affect cell migration at the applied concentration. Further scrutiny of the single cell migration by mean square displacement (MSD) vs. time interval in log-log plots revealed that the MSD of the T98G cells was not appreciably affected by 0.5% IPA (Fig. 4 B, first two panels). However, cell migration was hindered by treatment with 5 µM LIG as the slope of the MSD was clearly reduced to ∼ 0 (Fig. 4 B, the third panel). The histograms of the CCDs provide further insight in that the presence of 5 µM LIG for five hours noticeably reduced the occurrence of cell step sizes larger than 0.5 µm (Fig. 4 C, all panels and insets).

### LIG Treatment Changes the Expression Levels of Rho GTPases

Biophysical assessments provide direct and clear evidence for the LIG effect on T98G cell migration. We further investigated the molecular cause of the LIG effect by monitoring the expression levels of proteins directly associated with cell migration. Since the Rho GTPases, RhoA, Rac1 and Cdc42, are known as the full set of molecular switches that collectively govern cytoskeletal remodeling during cell migration, Western blotting against those three Rho GTPases was conducted to provide a molecular explanation for the observed LIG effect.

**Figure 5:**
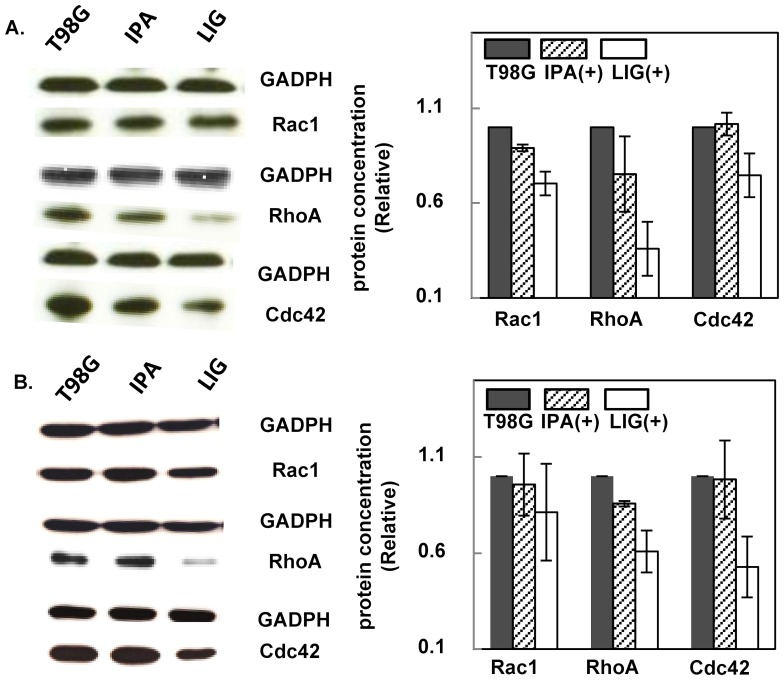
LIG treatment changes the expression level of the Rho GTPases in T98G cells. **A**) High concentration T98G cells were treated with 0.5% IPA or 5 µM LIG for 20 hours and then analyzed by Western blotting against RhoA, Rac1 and Cdc42. A housekeeping protein, GADPH, was also detected as a reference. The results were normalized using the control condition (no treatment) as reference. **B**) Similar tests were performed with low concentration T98G cells (single cell assay conditions) and 5 hours of incubation under 0.5% IPA or 5-µM LIG treatment. Error bars represent the standard error from two experiments.

The Western blotting results at high cell density, with or without 20-hour LIG incubation, were compared to probe the LIG effect on T98G cells in the wound-like gap closure process (Fig. 5 A). Samples at low density (single cell assay conditions), with or without 5-hour LIG incubation, were also studied (Fig. 5 B). The 5-hour incubation time was chosen because this is the time frame when single migrant cells were observed to leave the bulky cell cluster during the wound-like gap closure process. To make it easier to cross-compare results from these two conditions, Western blotting was also conducted at single cell concentrations with 20-hour LIG treatment (Fig. S3). The results elucidated that the expression levels of the three Rho GTPases were all modulated under LIG treatment and that RhoA was decreased the most. These results were in agreement with the conclusion drawn from the biophysical assays: the LIG treatment effect was due to the presence of LIG and not 0.5% IPA. Hence, the LIG effect to reduce T98G cell migration ability was confirmed.

## Discussion and Conclusion

This study demonstrates that Z-ligustilide (LIG), an extract from the essential oils of RAS, has a specific effect on GBM T98G cells to hinder cell migration. This conclusion is supported by consistent results from three distinct approaches. 1) The monitoring of the wound-like gap closure process with the consideration of apoptosis and changes in cell proliferation. 2) Direct single cell migration analysis, which supports the wound-like gap closure results and rules out cell-cell interactions as a cause of reduced migration. 3) The measurement of the expression levels of Rho GTPases, the three master regulators of cell migration. The wound-like gap closure assays also suggest that this effect is dose-dependent, which further strengthens our conclusion.

It has been reported that LIG has a cytotoxic effect on certain cell types, such as PC12, SH-SY5Y, HeLa, and C6, when the concentration of LIG is higher than 50 µM [13]. This study shows that LIG can significantly change the migration pattern of T98G cells at a concentration as low as 5 µM. Besides the identification of the new pharmaceutical effect of LIG treatment on T98G cells, this study also demonstrates that cell migration analysis can be a highly sensitive method for detecting cellular effects of potential drug candidates, which can be difficult to probe by endpoint assessment in pharmacology.

IPA is a non-polar solvent that is used to dissolve LIG in the mobile phase for HPLC to separate LIG from other RAS-extracted compounds [14]. LIG dissolved in IPA can be homogeneously distributed to cell media. However, our study shows that IPA alone could slightly alter the single cell migration pattern but not the wound-like gap closure process involving cell-cell interactions. Hence, the presence of IPA may directly affect cell behavior by hindering the cell migration capacity but this effect could be shielded by cell-cell interactions through signaling crosstalk. There is another possibility: the evaporation of IPA during the incubation period could also make the IPA’s effect inconsistent. Since the wound-like gap closure process takes much longer (more than 14 hours) than the single cell assay (only monitored for one hour during the fifth hour after adding IPA), the IPA’s effect on T98G cells could be gradually diminished and down-played during the time course of the wound-like gap closure process.

A single cell is subjected to fewer constraints when it attempts to change moving direction compared to cells in clusters. This freedom comes from not only the spatial availability but also, more predominantly, from the governance of cell polarity by cell-cell and cell-substrate interactions [12]. The signaling proteins involved in cell-cell interactions might also influence cell migration. Hence, the single cell migration analysis could provide more straightforward information regarding cell migration because several factors that could affect the wound-like gap closure process (i.e., cell-cell interactions, cell density and the rates of cell apoptosis and proliferation) could be excluded from single cell migration analysis. When the results from the wound-like gap closure process and single cell migration are compared, they suggest that the effect of LIG is significant.

The three Rho GTPases, RhoA, Rac1 and Cdc42, are known to be the molecular switches for the actin-cytoskeleton [11] remodeling. Our results further suggest that the LIG effects on cell activities are through fine-tuning the expression levels of Rho GTPases. It has been reported that it is the expression levels of Rho GTPases, but not their mutations, that govern change of cancer cell activities, such as metastasis. Furthermore, it has been shown that LIG can inhibit ERK-MAP kinase, which is an upstream regulator of RhoA and Rac1 in colon carcinoma cells [15], and suppress tumor necrosis factor-alpha (TNF-α), which can effectively activate RhoA and ROCK in human pulmonary microvascular endothelial cells [16]. Moreover, in PC12 cells, LIG can activate PI3K/Akt pathways [17], which directly up-regulate Rho GTPases.

The study provides a clear perspective that explains the inhibitive effect of Z-ligustilide on T98G cell migration and suggests that Z-ligustilide may be an effective agent for protecting the central nervous system against GBM diseases, partially through the modulation of Rho GTPase expression. Furthermore, this study also shows that cell migration can be used to understand the outcome of altered Rho GTPase activity as a potential approach for drug screening.

## Supporting Information

Figure S1 LIG solution reduced T98G cells’ migration in a dose-dependent manner. The wound-like gap closure process of T98G cells under mock, 0.5% isopropanol (IPA), 2.5 µM, 5 µM, 10 µM, and 25 µM LIG (top to bottom) is presented at 0^th^, 3^rd^, 8^th^, 14^th^ and 18^th^ hour until the mock T98G totally healed. Yellow lines indicate the approximate boundary between the cell-free wound region of images and the region corresponding to migrating cells. Scale bar: 200 µm.
(TIFF)

Figure S2 LIG at tested concentrations does not affect T98G apoptosis. Three groups of experiments were conducted: A) T98G cells, treated with and without 8 μΜ camptothecin, were tested through flow cytometry to achieve the selection of population gate and apoptosis gate ((P3: population gate for selecting cells to be analyzed R1: apoptosis gate for selecting apoptotic cells). B) High density T98G cells and C) Low concentration T98G without any cell-cell interactions were each incubated with 0.5% IPA and 5 µM LIG solution, respectively. LIG did not lead to any difference in the number of apoptotic cells. Each experiment was conducted in duplicate.
(TIFF)

Figure S3 LIG treatment reduced the expression levels of the Rho GTPases in T98G cells. T98G cells were loaded at the conditions for single cells and incubated with 0.5% IPA or 5 µM LIG for 20 hours and then analyzed by Western blotting against RhoA, Rac1 and Cdc42 (a housekeeping protein, GADPH, was also detected as a reference to to calibrate the relative amount of RhoA, Rac1 and Cdc42.). The results were normalized using the control condition (no treatment) as reference.
(TIFF)
