# Supplementary figures and images for: The Effect of Z-Ligustilide on the Mobility of Human Glioblastoma T98G Cells
Source: PLoS One. 2013 Jun 21;8(6):e66598. doi: 10.1371/journal.pone.0066598 (PMC3689746; doi:10.1371/journal.pone.0066598)

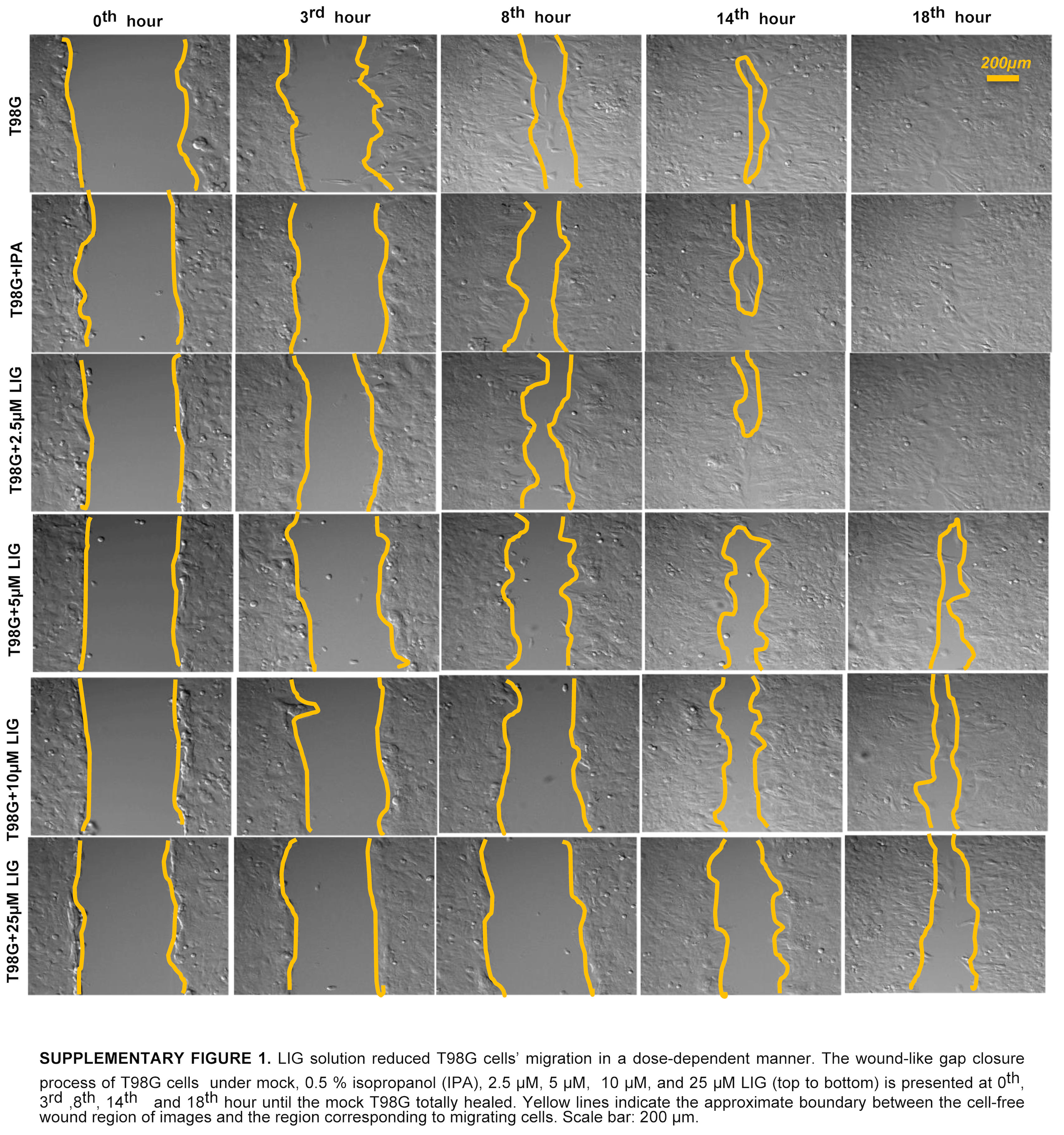

Supplement: Figure S1 — LIG solution reduced T98G cells’ migration in a dose-dependent manner. The wound-like gap closure process of T98G cells under mock, 0.5% isopropanol (IPA), 2.5 µM, 5 µM, 10 µM, and 25 µM LIG (top to bottom) is presented at 0th, 3rd, 8th, 14th and 18th hour until the mock T98G totally healed. Yellow lines indicate the approximate boundary between the cell-free wound region of images and the region corresponding to migrating cells. Scale bar: 200 µm. (TIFF) [file pone.0066598.s001.tiff]

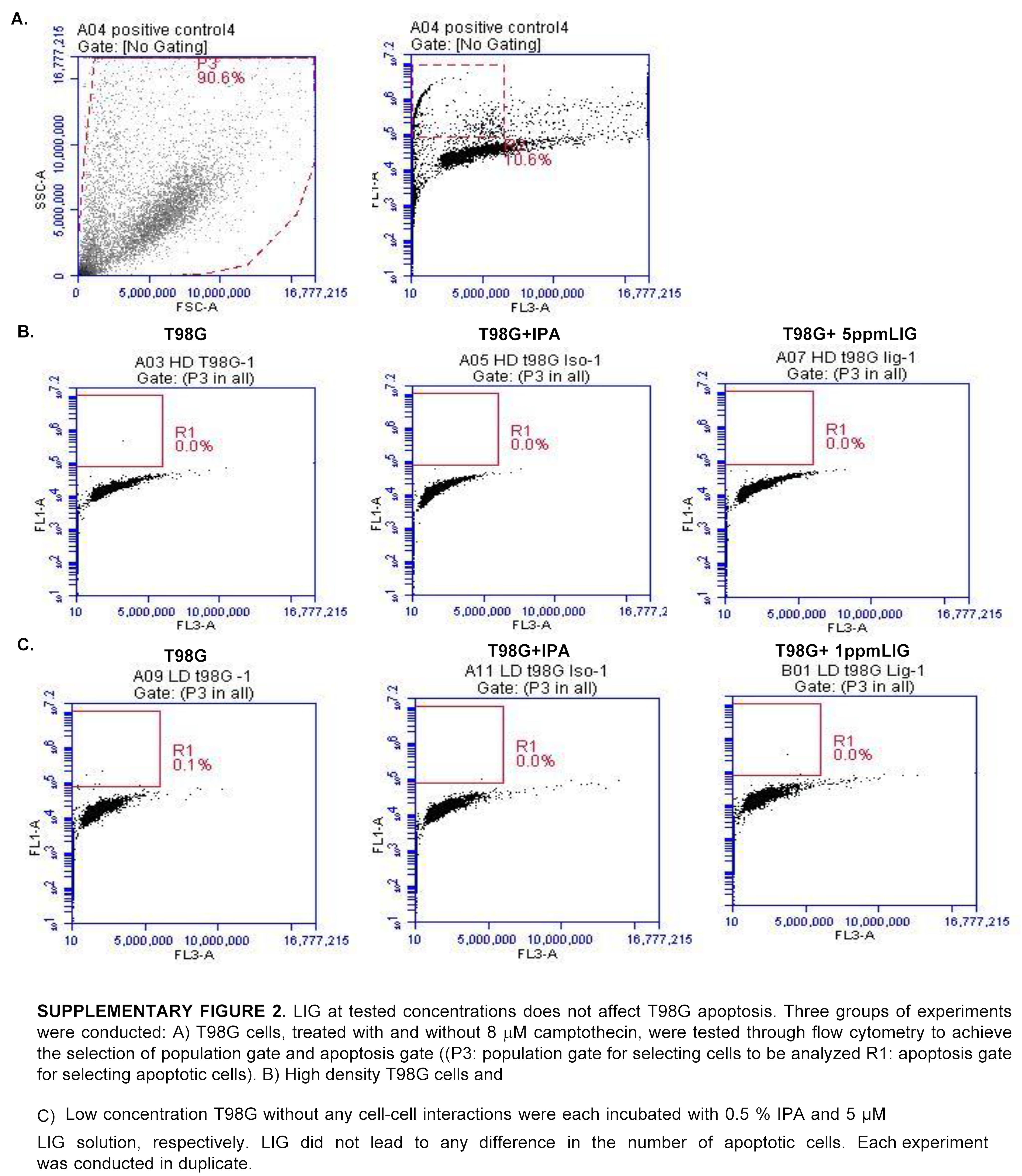

Supplement: Figure S2 — LIG at tested concentrations does not affect T98G apoptosis. Three groups of experiments were conducted: A) T98G cells, treated with and without 8 μΜ camptothecin, were tested through flow cytometry to achieve the selection of population gate and apoptosis gate ((P3: population gate for selecting cells to be analyzed R1: apoptosis gate for selecting apoptotic cells). B) High density T98G cells and C) Low concentration T98G without any cell-cell interactions were each incubated with 0.5% IPA and 5 µM LIG solution, respectively. LIG did not lead to any difference in the number of apoptotic cells. Each experiment was conducted in duplicate. (TIFF) [file pone.0066598.s002.tiff]

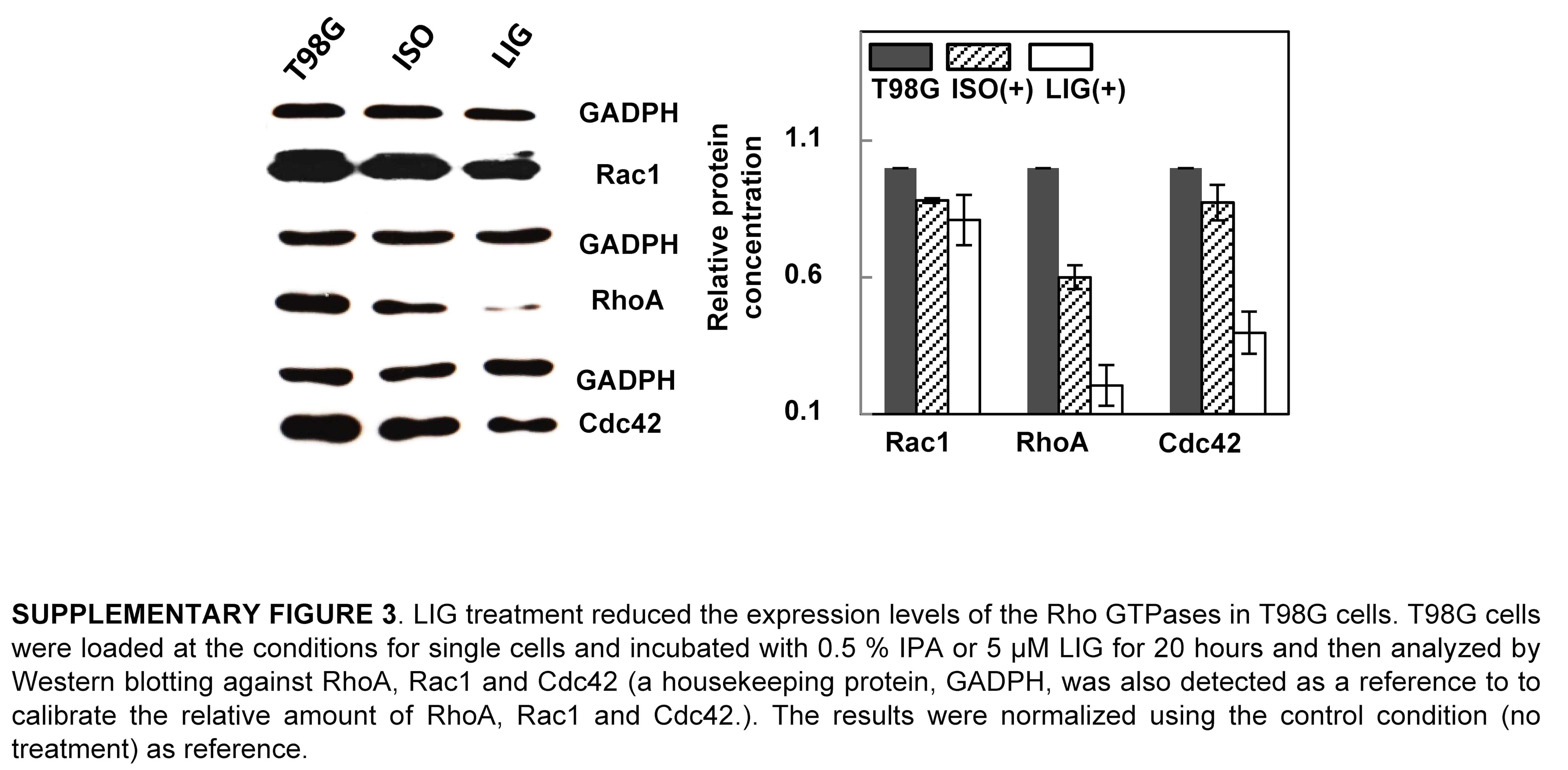

Supplement: Figure S3 — LIG treatment reduced the expression levels of the Rho GTPases in T98G cells. T98G cells were loaded at the conditions for single cells and incubated with 0.5% IPA or 5 µM LIG for 20 hours and then analyzed by Western blotting against RhoA, Rac1 and Cdc42 (a housekeeping protein, GADPH, was also detected as a reference to to calibrate the relative amount of RhoA, Rac1 and Cdc42.). The results were normalized using the control condition (no treatment) as reference. (TIFF) [file pone.0066598.s003.tiff]
